# Estimating hearing aid fitting presets with machine learning–based clustering strategies

**DOI:** 10.1121/10.0007149

**Published:** 2021-11-19

**Authors:** Chelzy Belitz, Hussnain Ali, John H. L. Hansen

**Affiliations:** Center for Robust Speech Systems, The University of Texas at Dallas, Richardson, Texas, 75075 USA

## Abstract

Although there exist nearly 35 × 10^6^ hearing impaired people in the U.S., only an estimated 25% use hearing aids (HA), while others elect not to use prescribed HAs. Lack of HA acceptance can be attributed to several factors including (i) performance variability in diverse environments, (ii) time-to-convergence for best HA operating configuration, (iii) unrealistic expectations, and (iv) cost/insurance. This study examines a nationwide dataset of pure-tone audiograms and HA fitting configurations. An overview of data characteristics is presented, followed by use of machine learning clustering to suggest ways of obtaining effective starting configurations, thereby reducing time-to-convergence to improve HA retention.

## 1. Introduction

Studies suggest that mild to severe hearing impairment causes adverse effects on quality of life and that these effects are largely reversible through the use of HAs.^1–3^ However, a 2008 survey found that of the 34.25 × 10^6^ people in the U.S. who reported experiencing hearing difficulty, only 24.6% owned HAs.^4^ Of those HA owners, many choose not to use their HAs at a rate of 4.2%–24% reported in various international surveys.^5–7^

Several of the barriers to obtaining an HA include cost, lack of insurance coverage, and the need for repeat visits for customized fittings.^8^ Among HA owners who do not use them, some of the most prevalent reasons cited across studies are reported low HA value and issues with fit or comfort of the HA.^5^ Low HA value is associated with several factors, including issues with noise, difficulty in human adjustment to HA use, and the need for repeated clinical visits for fine-tuning of HA system settings to achieve the best programming for an individual.

**Fig. 1.**
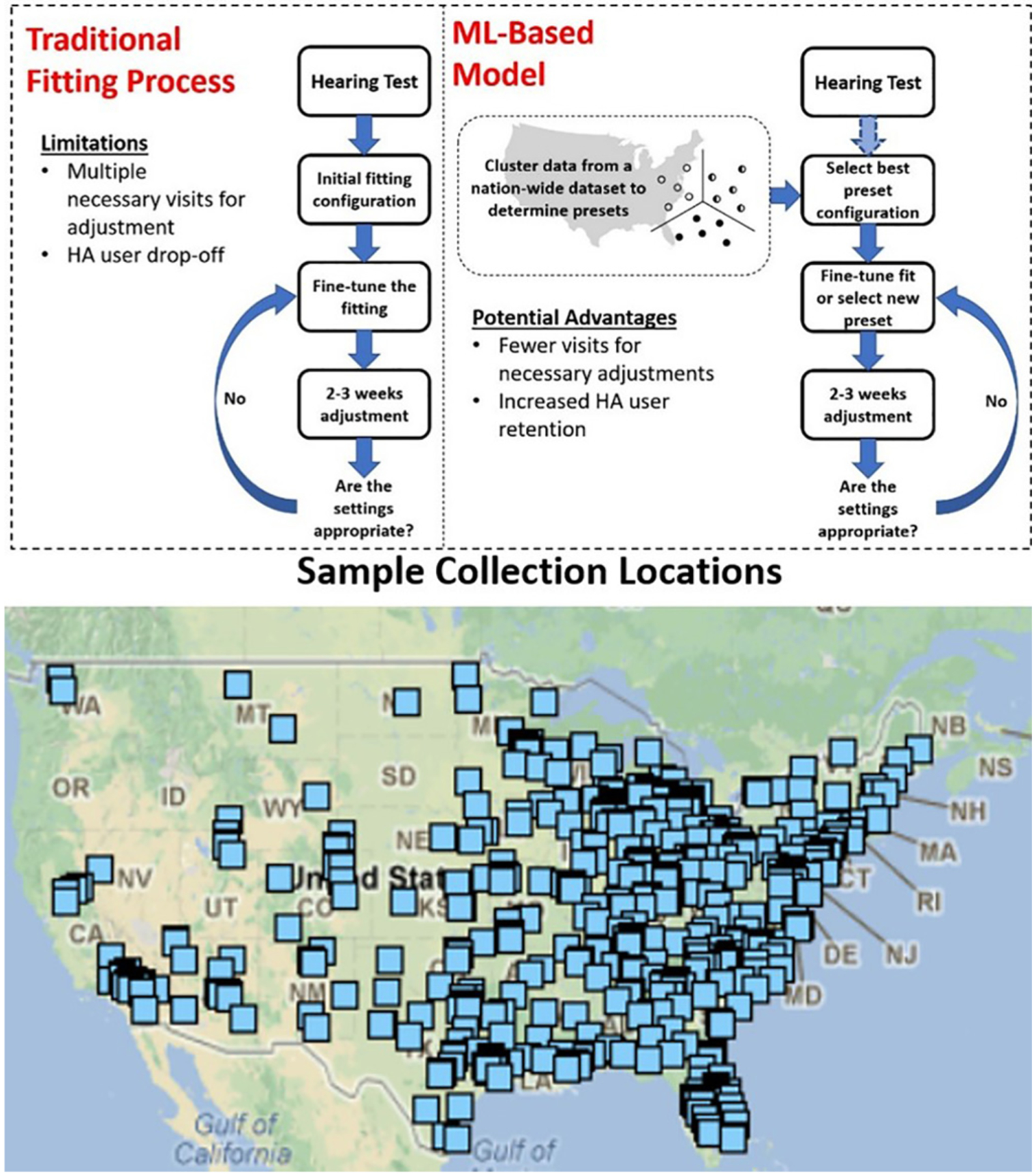
An overview of the procedure proposed for establishing HA starting configurations and a map showing a sample of collection locations across the United States.

It is suggested that by reducing the amount of necessary adjustment, the HA programming process can be better streamlined to make amplification products, such as HAs, more readily accessible to more individuals and to improve HA retention. Furthermore, in the U.S., the Over-The-counter Hearing-Aid Act of 2017 has sparked interest in investigations of HA configurations for use in more cost-effective amplification products.^9^ This study, therefore, explores a massive nationwide corpus to research potential ML methods to establish better starting configurations for amplification products. Figure 1 shows an overview of the suggested procedure, suggesting the potential advantage of the incorporation of a ML-based model.

## 2. Database

### 2.1 Structure and content

Linked nationwide databases from a single HA manufacturer including client audiograms and HA fitting settings were studied. A great deal of database variability existed from the estimated 500 locations from across the U.S.

Eleven place-holder frequencies were listed in the database for pure-tone audiometry evaluation: 125, 250, 500, 750, 1000, 1500, 2000, 3000, 4000, 6000, and 8000 Hz.

Approximately 4.3 × 10^6^ entries are included in the audiogram database, including entries from three audiogram types. This includes uncomfortable loudness level (UCL), bone conduction level (BCL), and hearing threshold level (HTL) for left and for right ears. The majority of clients have entries for both ears, with HTL audiogram type representing the most common of all entries (36.37%). Considering this, the audiogram database includes just under 800 000 unique clients.

**Fig. 2.**
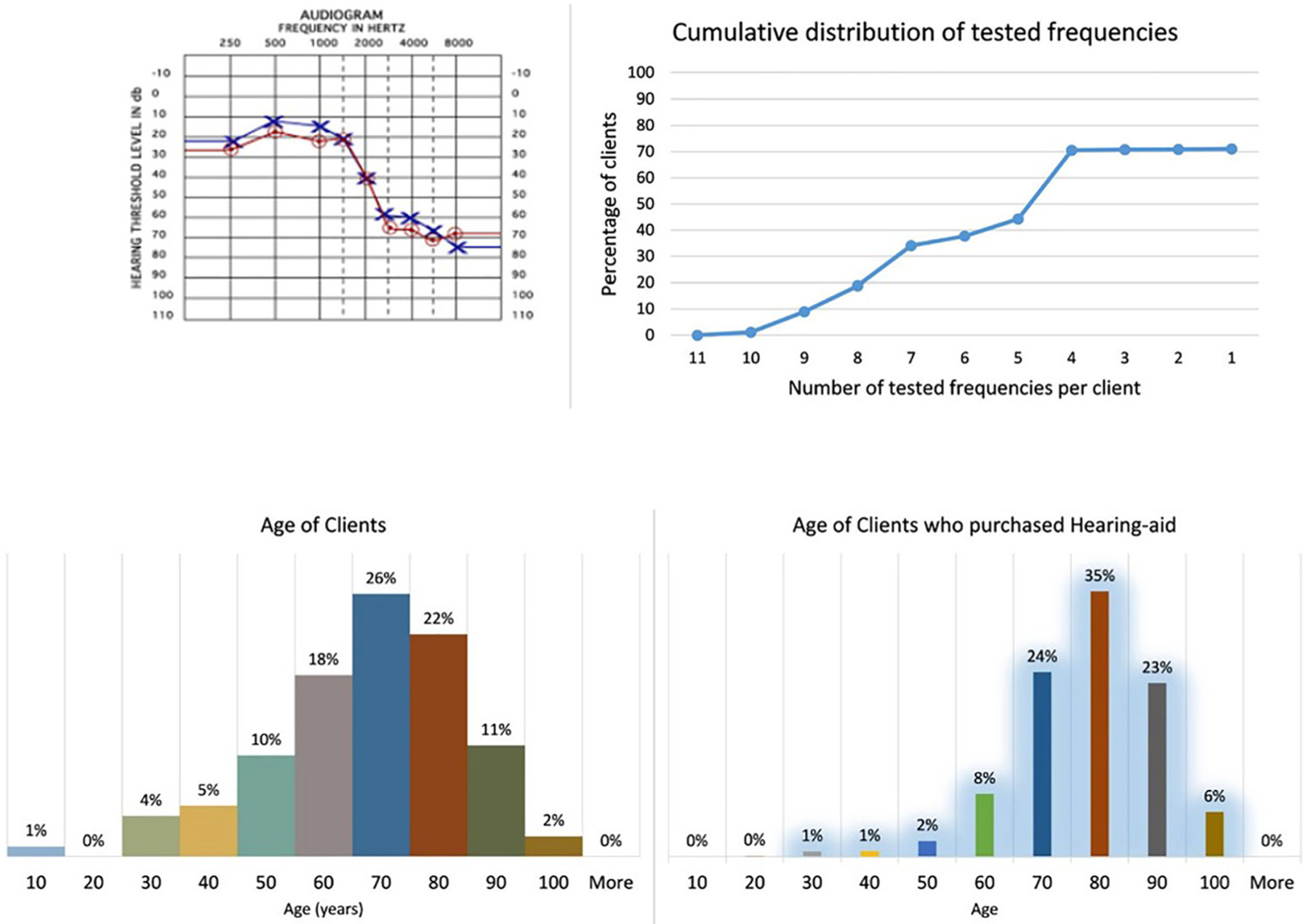
An overview of the database contents. Upper left: A sample audiogram. Upper right: Cumulative distribution of the audiometry data for the number of test frequencies. Lower: Age distribution of all unique clients within the database.

Of all audiogram entries included within the database, only 71% listed a value for at least one of the test frequencies. Figure 2 shows the resulting corpus based cumulative distribution of valid audiometry data for the tested frequencies.

Nearly all entries that listed at least one test frequency also listed the audiometry data for at least four test frequencies. In all, 70.34% of the total client entries included audiometry data for the four core frequencies: 500, 1000, 2000, and 4000 Hz. Taking this into account, the majority of the analysis here relies on HTL audiograms using only the values for these four core frequencies.

For the other half of this massive user corpus, the HA fitting database reflected a similar structure to the audiogram database. As in the audiogram database, eleven placeholder frequencies were available to record device gain. The fitting database includes notably fewer entries overall, with approximately 1.9 × 10^6^ entries present.^10^

In this database, seven fitting types were included with profiles created for each client in each of these database entries. This analysis focuses predominantly on comfort target, a measure intended to keep users of amplification devices in a comfortable hearing range by ensuring that high intensity sounds are not overly amplified to the point of nearing the pain threshold while keeping low intensity sounds amplified within the audible range of a user.

In all, roughly 16% of unique audiograms had a valid entry for the associated fitting data. This resulted in just over 90 000 audiogram data to HA fitting data pairs comprising this study’s core dataset for research.

### 2.2 Demographics

The dataset examined includes a total of 855 835 client entries from more than 500 unique locations throughout the U.S. Of these entries, 77% include valid date-of-birth with 65 y as the median client age, and 62.7 y as the mean age. The age distribution for clients with valid date-of-birth entries is shown in Fig. 2. Notably, more than half (67%) of client entries fall within the 50–80 y age range.

Further examination of the client database shows that of all data entries, only 20.67% (176 865 entries) were identified as having purchased a HA. The age distribution for clients who purchased HAs is shown in Fig. 2. The median age of clients who purchased a HA was nearly 10 y greater than all clients tested for hearing (74 y versus 65 y). The mean age was 72.7 y.

### 2.3 Variability

It is important to note that the data from both the audiogram database and the HA fitting database came from a vast number of individual sites located throughout the U.S. Data were collected by individuals of varied levels of training and experience, and so a level of data variability is involved in the data collection. This includes the potential for human error and issues caused by manual data entry stemming from the more than 500 U.S. data collection sites.

## 3. Proposed solution: Establishing initial HA configurations

This study combines unsupervised and supervised machine learning methods to determine a small set of initial HA configurations that can act as preliminary HA settings derived from audiogram hearing results. The proposed solution treats the problem as a two-phase evaluation. The first step involves determining a limited number of effective preset configurations to represent the fitting targets. To determine the similarities among the fitting targets, various audiogram clustering methods are examined. The centroids of the audiogram clusters are then selected as the representative fitting targets which act as preset starting configurations for a HA.

It is suggested that by starting with a more effective user setting, it will require fewer adjustments/visits to converge to a better overall suited HA setting for the individual. The second step of the problem is to map pure-tone audiograms to the selected preset HA configurations by assigning a classification to each audiogram, based on results using comfort target clustering. Each audiogram is assigned to the same cluster as its associated comfort target. Various classification methods are considered for assigning each audiogram to a preset configuration based on the cluster labels.

After administering a hearing evaluation, a client may be suggested to consider an amplification product with a user targeted setting from one of the limited number of preset starting configurations. From there, the HA may be adjusted by the audiologist/specialist to better suit the needs of the individual.

## 4. Comfort target clustering

One method for providing a small set of preconfigured HA settings is to create meaningful data clusters. For each cluster, one point is selected as an initial configuration. Here, the point selected is the centroid for each cluster. The goal is to minimize the amount of client/audiologist–specialist adjustment between the initial configuration selected and each point within the cluster, considered to be the optimal configuration for a particular HA user. To evaluate the total adjustment, the mean absolute error (MAE) is calculated for each cluster, using the selected initial configuration as the estimated value, along with using each of the data points associated as the true value. Eq. (1) shows the calculation for the average adjustment per frequency per cluster. Here, ***y***_***i***_ is the true value of the comfort target array, ${\hat{y}}_{i}$ is the estimated value (here, the centroid of the associated cluster is used), and n is the number of comfort target arrays. Here, this value is considered the average total adjustment,

(1) $$\text{MAE}=\frac{1}{n}\sum_{i=1}^{n}|y_{i}-{\hat{y}}_{i}|.$$

Using this evaluation method, it is anticipated that distance-based clustering algorithms should be the most appropriate. In particular, k-means, which iteratively reduces the distance between cluster centroids and points within a cluster, should perform best. For an initial evaluation, a small sample of the comfort target data (10%) was evaluated using three different distance-based clustering algorithms. The MAE of each is shown in Table 1.

### 4.1 K-means

**Fig. 3.**
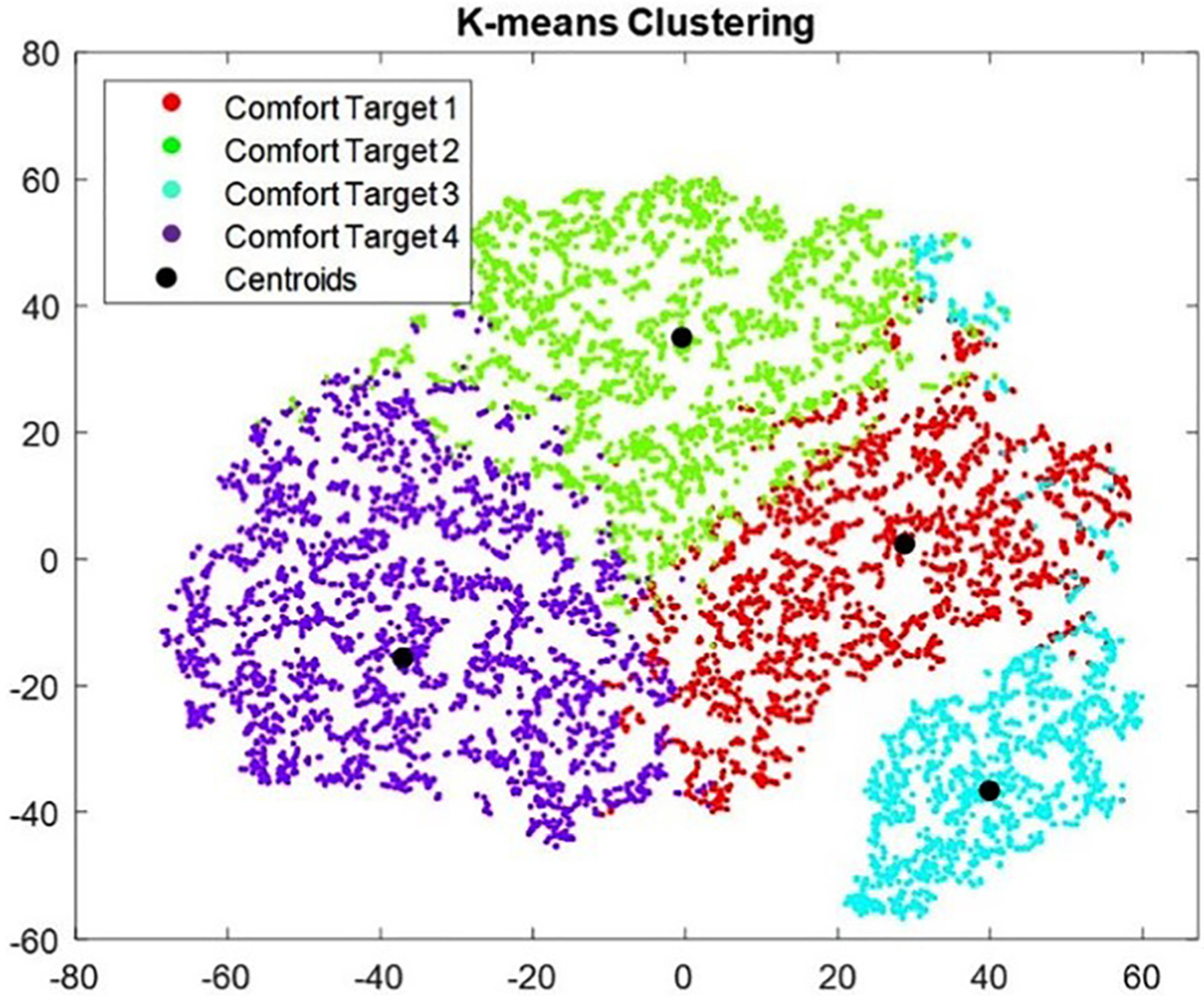
The comfort target clusters created using k-means clustering.

From the initial evaluation for the clustering methods with 10% of the data, k-means performs the best given the criteria laid out. Applying k-means clustering to the entire HA comfort target data set results in the clusters illustrated in Fig. 3. T-SNE is used to project the data into a two-dimensional space for visualization.^11^

Further analysis revealed that by considering the standard deviation of each dimension independently, an average of 26.92% of comfort targets considered in the dataset are within one-standard deviation of the centroid value of their respective clusters. This shows that a large portion of comfort targets are relatively close to a potential initial starting centroid. Table 2 summarizes the results for each proposed starting cluster.

### 4.2 Number of clusters

**Fig. 4.**
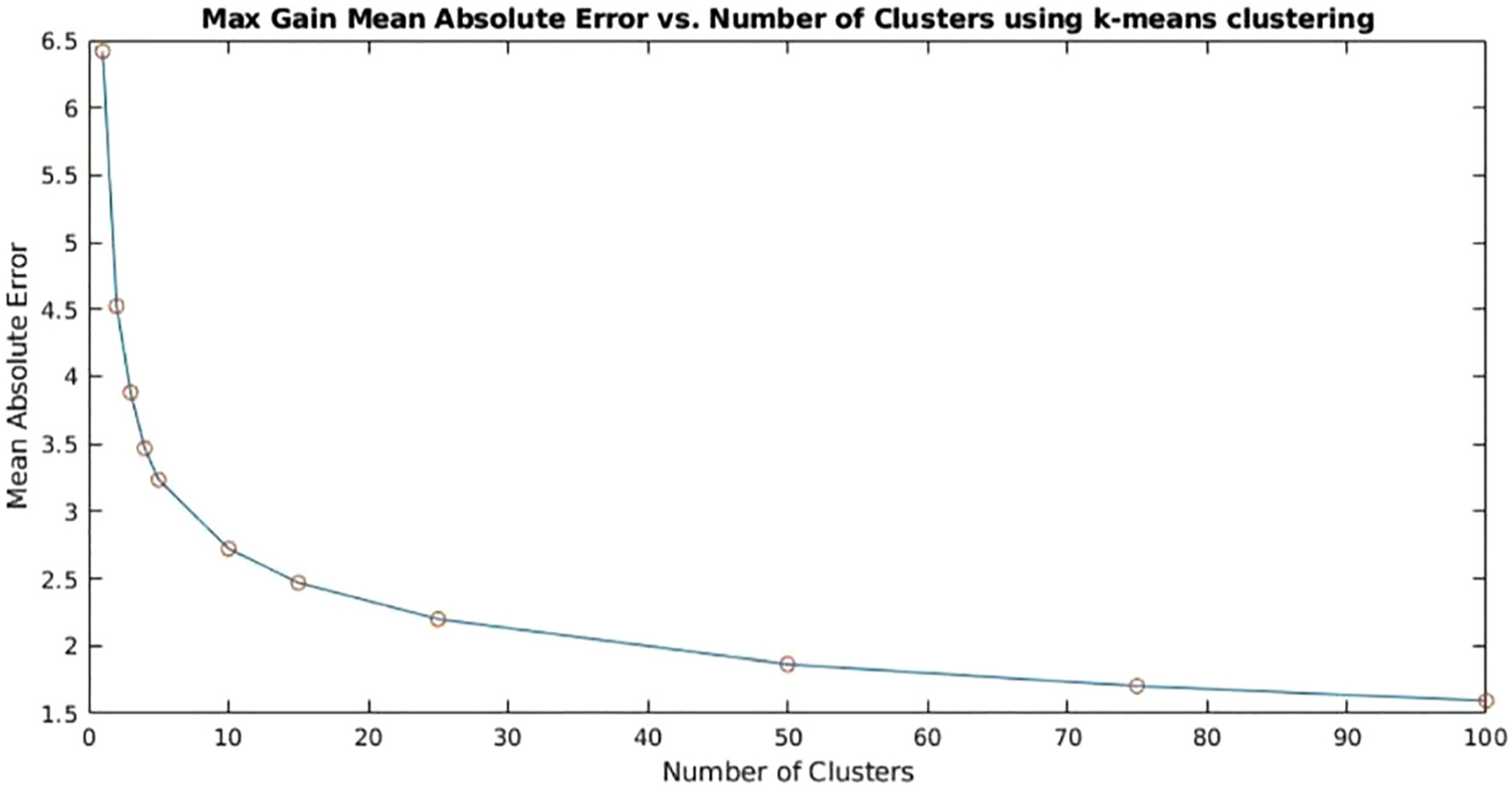
A plot of the MAE versus the number of clusters. It may be noted that increasing the number of clusters results in an exponential decrease in the MAE.

Using the evaluation procedure described, it is clear that increasing the number of potential clusters should always yield a lower necessary adjustment from the centroids to each of the points assigned to a cluster. As the number of centroids approaches the number of total data points, the overall MAE will converge to zero. This trend is seen in Fig. 4, which plots the MAE as the number of clusters k is increased for a subsection of the data.

It may be noted that the largest drop in MAE occurs between increasing from one to two clusters. From there, the change in MAE drops with each additional cluster. This, in turn, begets the question of how many clusters is appropriate for final evaluations? Selecting too small a number results in a greater need for adjustment between the selected pre-configurations and the final ideal configurations. However, selecting too large a number results in too many assignment possibilities which increases the potential for misassignment of points to a neighboring cluster, producing a final solution which is not feasible. The number of configurations must therefore consider effective representation of the points within a cluster while considering the potential introduction of misassignments as well as physical realizability. Particularly, if the desired result is to create amplification devices with pre-configured settings stored on the device initially, the number of preset configurations would be limited by hardware.

In this evaluation, the selected preset value of k is 4, which is selected to intentionally create a very small number of presets while achieving effective acoustic space coverage. However, it should be noted that an elbow occurs at 10 clusters, indicating diminished returns as we increase k above 10. So, as a best practice for effective acoustic space coverage, 10 clusters is the upper value recommendation.

## 5. Mapping

**Fig. 5.**
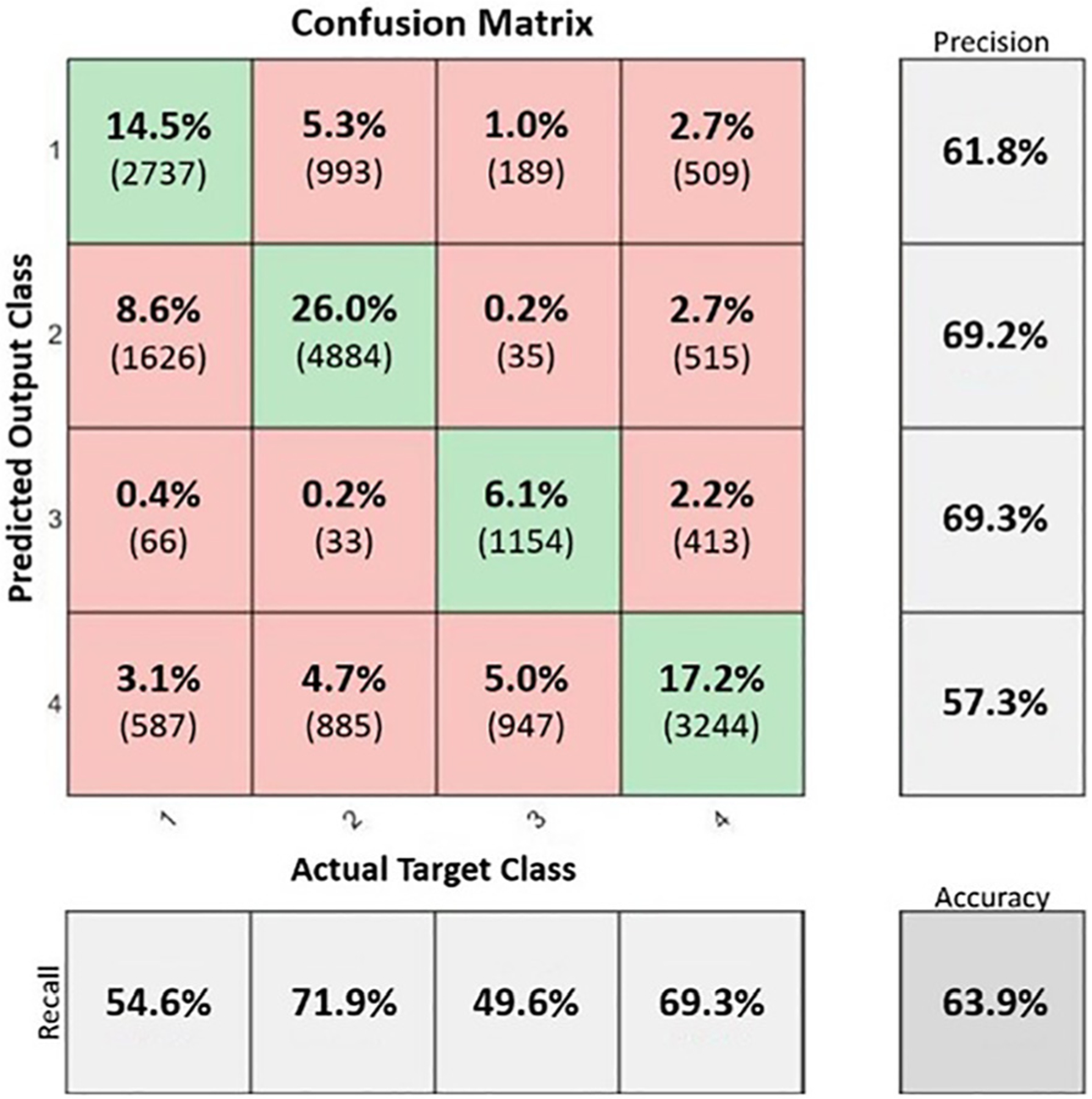
A confusion chart showing the performance of the classification model.

Taking comfort target fitting assignment as the output classification label, and using the audiogram as the input data, the audiometry data were assigned to one of the clusters. Taking 20% of the total +90 000 complete audiograms with associated fittings and setting them aside for testing, a test accuracy of approximately 64% was achieved using simple classification methods. This relatively low accuracy is likely because although clustering was able to create distinct regions in the comfort target space, the associated audiograms within each cluster were highly overlapped. Figure 5 shows the overall performance of the model with centroid classification accuracies (percent and raw counts) along with centroid misclassifications. Since the goal is to identify an effective starting location, by taking a top two accuracy for classification, the resulting overall test accuracy climbs to 92.70%. This effectively shows that it is possible to eliminate two of the four potential starting locations, achieving a better start location for HA fitting.

## 6. Conclusion

Unsupervised learning methods allow for the exploration of data to derive similarities. To reduce HA fitting time-to-convergence for adjustment between an initial setting and a user’s final optimal setting, this study focused on algorithms that emphasize grouping based on human hearing audiogram frequency results to HA comfort level distance. K-means, in particular, was shown to have particular advantages running over a large data set. By selecting the system setting centroid values to represent all points within the clusters, it was possible to determine general settings that best represent large sections of data, allowing for the selection of a limited number of pre-configurations that are representative of the data as a whole. While the exact number of presets here was four, other set sizes are possible, depending on the range of HA design parameters considered and specific manufacturers. The study here has demonstrated that this limited selection has allowed for devices to be preprogrammed, providing more reasonable hearing based start locations, thus further increasing the success and accessibility of HA devices to those who need them, as well as potentially improving adoption and use for the benefit of hearing-impaired subjects.

## Figures and Tables

**Table 1.** A summary of the average total adjustment for each clustering algorithm tested.

Algorithm	MAE
Ward	5.15
Birch	5.36
K-means	4.92

**Table 2.** A summary of the percent of points falling within 1–3 standard deviations considered per dimension.

Cluster	1 STD (%)	2 STD (%)	3 STD (%)
Cluster target 1	22.64	22.64	97.61
Cluster target 2	29.03	84.90	97.78
Cluster target 3	22.06	83.18	97.15
Cluster target 4	33.95	88.39	97.43
